# Paranodal Axoglial Junctions, an Essential Component in Axonal Homeostasis

**DOI:** 10.3389/fcell.2022.951809

**Published:** 2022-07-06

**Authors:** Tomoko Ishibashi, Hiroko Baba

**Affiliations:** ^1^ Department of Functional Neurobiology, Tokyo University of Pharmacy and Life Sciences, Hachioji, Japan; ^2^ Department of Occupational Therapy, Faculty of Rehabilitation, Niigata University of Health and Welfare, Niigata, Japan

**Keywords:** paranodal junction, myelin, Purkinje, calcium, IP3R1

## Abstract

In vertebrates, a high density of voltage-gated Na^+^ channel at nodes of Ranvier and of voltage-gated K^+^ channel at juxtaparanodes is necessary for rapid propagation of action potential, that is, for saltatory conduction in myelinated axons. Myelin loops attach to the axonal membrane and form paranodal axoglial junctions (PNJs) at paranodes adjacent to nodes of Ranvier. There is growing evidence that the PNJs contribute to axonal homeostasis in addition to their roles as lateral fences that restrict the location of nodal axolemmal proteins for effective saltatory conduction. Perturbations of PNJs, as in specific PNJ protein knockouts as well as in myelin lipid deficient mice, result in internodal axonal alterations, even if their internodal myelin is preserved. Here we review studies showing that PNJs play crucial roles in the myelinated axonal homeostasis. The present evidence points to two functions in particular: 1) PNJs facilitate axonal transport of membranous organelles as well as cytoskeletal proteins; and 2) they regulate the axonal distribution of type 1 inositol 1,4,5-trisphosphate receptor (IP_3_R1) in cerebellar Purkinje axons. Myelinated axonal homeostasis depends among others on the state of PNJs, and consequently, a better understanding of this dependency may contribute to the clarification of CNS disease mechanisms and the development of novel therapies.

## Introduction

**FIGURE 1:**
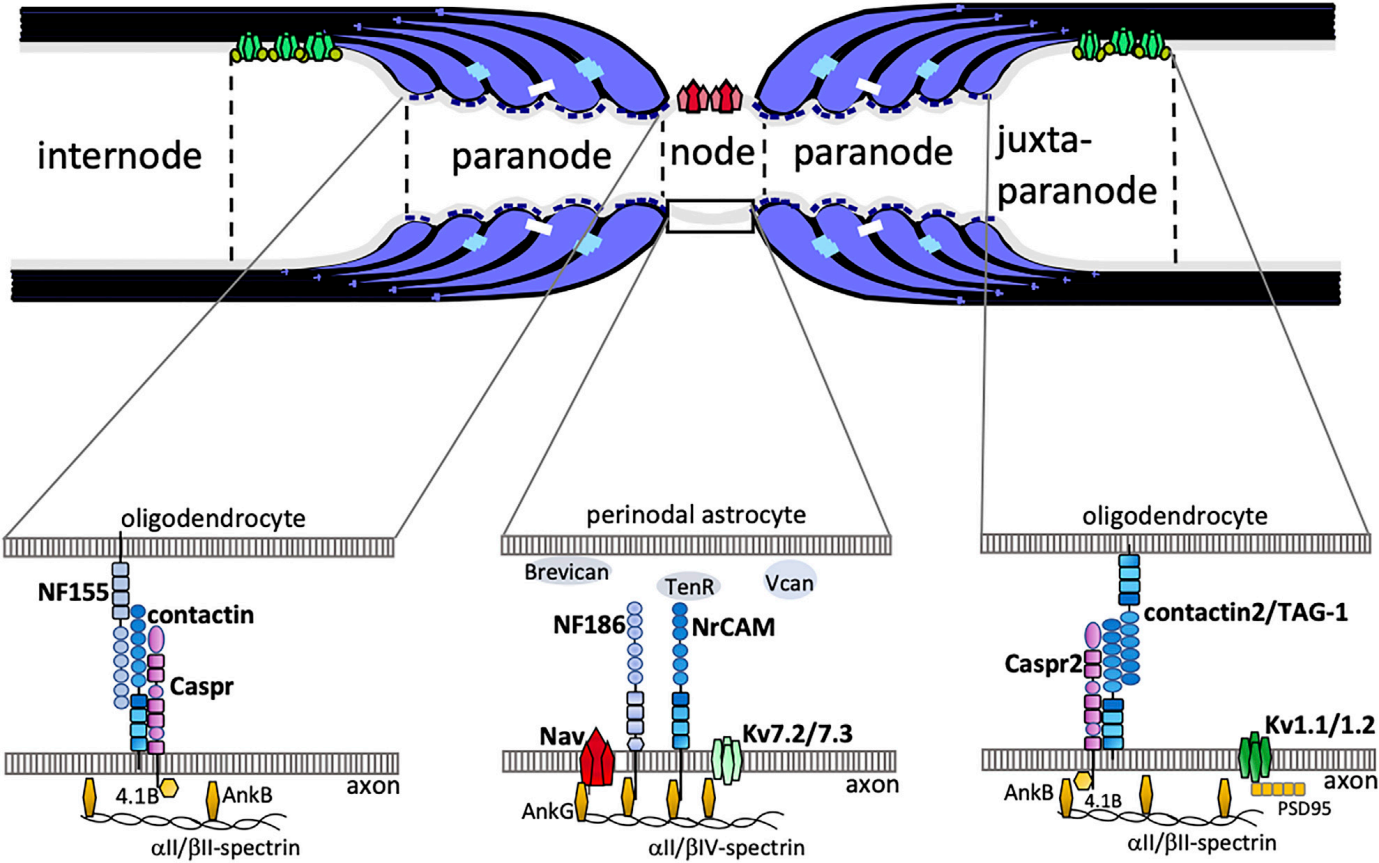
The schematic drawing shows junctional complexes between oligodendrocytes and axons in myelinated axons. The nodal axolemma with voltage-gated Na^+^ channels (Nav) has concentrations of neurofascin 186 (NF186) and neuronal cell adhesion molecule (NrCAM) belonging to the L1-family of CAMs at the node of Ranvier. The cytoplasmic region of axonal NF186 and NrCAM binds ankyrinG (AnkG), which anchors the nodal complex to βIV-spectrin and to the actin cytoskeleton. AnkG enables the clustering of Nav and Kv7.2/7.3 channels. Extracellular matrices such as Brevican, Versican (Vcan), and TenascinR (TenR) are surrounding the nodes. At the paranode, the cis-complex of Caspr and contactin interacts in trans with NF155 on the paranodal myelin loop. This complex is stabilized by protein 4.1B which co-localizes with AnkB, aII/bII-spectrin. At the juxtaparanode, Shaker-type voltage gated K+ channels Kv1.1/1.2 form clusters. A complex of contactin2 (also known as TAG-1) and Caspr2 is implicated in the formation of juxtaparanodes.

Myelin is a membrane structure that developed in the evolution of vertebrates (Boullerne, 2016; Salzer and Zalc, 2016). In the developmental stage of the central nervous system, axons become myelinated through wrapping by oligodendrocytes. The resulting myelinated axons have distinct specialized regions: nodes of Ranvier, paranodes, juxtaparanodes, and internodes (Figure 1). At the nodes of Ranvier there is a high density of voltage-gated Na^+^ channels (Nav1.2 and Nav1.6) that conduct inward depolarizing currents, whereas juxtaparanodal K^+^ channels (Kv1.1 and Kv1.2) maintain electrical stability and polarization (Ritchie and Rogart, 1977; Caldwell et al., 2000). As a result, rapid conduction of nerve impulses, known as saltatory conduction, is achieved by action potentials leaping along myelinated nerves via the nodes of Ranvier (Tasaki, 1939; Huxley and Stampfli, 1949). A recent study by Kanda et al. showed that two-pore-domain potassium (K2P) channels, TREK-1 and TRAAK, are also clustered at the nodes of Ranvier and that these are required for rapid action potential regeneration (Kanda et al., 2019). At the paranodes adjacent to the nodes of Ranvier, there are paranodal axoglial junctions (PNJs) that are characterized by intermittent densities associated with the outer leaflet of the axolemma, called transverse bands (Peters, 1966; Rosenbluth, 1976), and myelin loops that attach to the axonal membrane (Rosenbluth, 1999). Three paranode-specific cell adhesion molecules are primarily required for the formation of PNJs: contactin (Rios et al., 2000; Bhat et al., 2001; Boyle et al., 2001) and contactin-associated protein (Einheber et al., 1997; Peles et al., 1997) on the side of the axonal membrane, and Neurofascin-155 on the side of the glial membrane (Tait et al., 2000; Sherman et al., 2005). Autoantibodies against Neurofascin-155 and contactin have been found in patients with neuropathy (Labasque et al., 2014; Koike et al., 2017; Vallat et al., 2017). Koike and colleagues demonstrated that these antibodies induced paranodal axoglial detachment, resulting in aberrant nerve conduction and axonal damage (Koike et al., 2017). Anti-Neurofascin-155 antibody is also present in patients with combined central and peripheral demyelination (CCPD; Kawamura et al., 2013; Kira, 2021).

The morphological structure of the nodes of Ranvier and their adjacent paranodes suggests that nodal formation and maturation depend on the state of the PNJs. In fact, it has been reported that nodal Na^+^ channel clustering depends on paranodal axoglial contact in the developing CNS (Rasband et al., 1999). These paranode-dependent clusterings of nodal Na^+^ channels require axonal βII spectrin, which is concentrated at the paranodes (Ogawa et al., 2006; Amor et al., 2017). Zhang et al. also reported that the accumulation of Neurofascin-186, a key adhesion molecule of nodes, is regulated by PNJs (Zhang et al., 2020). Additionally, PNJs are required for the maintenance of Na^+^ channel clustering and nodal maturation (Ishibashi et al., 2002; Ishibashi et al., 2003; Rasband et al., 2003). More recently, Elazar et al. demonstrated that coordinated action of internodal (Cadm4 and MAG) and paranodal (Caspr) cell adhesion molecules is required for accurate myelination (Elazar et al., 2019a; Elazar et al., 2019b). Hence, the PNJs play several pivotal roles—among others, they promote node formation and are anchored to the axonal cytoskeleton, thus serving as a fence that limits the lateral movement of axolemmal proteins and keeps them in place (Rasband et al., 1999; Amor et al., 2014, 2017; Brivio et al., 2017). Furthermore, there is growing evidence that the PNJs contribute to the maintenance of functional, myelinated axons and of axonal homeostasis (Pillai et al., 2009; Rosenbluth, 2009; Ohno et al., 2011; Ishibashi et al., 2015). Numerous reviews (Rosenbluth, 2009; Faivre-Sarrailh et al., 2013; Rosenbluth et al., 2013; Arancibia-Carcamo and Attwell, 2014; Susuki et al., 2016; Faivre-Sarrailh, 2020) have discussed the identities and roles of the ever-increasing number of molecules associated with the adhesion molecules at the paranodes, as well as voltage gated Na^+^ channels at the nodes and voltage gated K^+^ channels at the juxtaparanodes. This review focuses on the significance of PNJs in the facilitation of axonal transport and in cerebellar Purkinje axonal homeostasis.

## PNJs: A Key Facilitator in Axonal Transport

Neurons are highly polarized cells with elongated axons that can reach a considerable distance from the perikaryon. In the axon, membranous organelles, molecular complexes, and cytoskeletal components of the axoplasm (such as microtubules and neurofilament) are transported by either fast or slow transport from one end of the axon to the other (Grafstein and Forman, 1980; Hirokawa, 1997). It is, therefore, essential for axonal polarity and axonal stabilization to maintain accurate axonal transport (Sleigh et al., 2019; Guillaud et al., 2020). Axonal mitochondrial dynamics have been studied especially intensively under both physiological and pathological conditions (Andrew et al., 2006; Kiryu-Seo et al., 2010; Chiu, 2011; Zambonin et al., 2011; Zhang et al., 2012; Itoh et al., 2013; Ohno et al., 2014; Sorbara et al., 2014; Joshi et al., 2015; Joshi et al., 2019; Kiryu-Seo and Kiyama, 2019).

Impairment of axonal transport precedes the axonal degeneration observed in demyelinating diseases, such as multiple sclerosis (MS) and amyotrophic lateral sclerosis (ALS), and is also a common hallmark in various neurodegenerative diseases (Coleman, 2005; Morfini et al., 2009; Sorbara et al., 2014; Sleigh et al., 2019; Guillaud et al., 2020). Altered axonal transport and focal axonal swelling formation with mitochondrial alterations have been reported in several knockout mice ablating specific myelin proteins (Griffiths et al., 1998; Lappe-Siefke 2003; Edgar et al., 2004), in dysmyelinating mouse *Shiverer* (Joshi et al., 2015; Joshi et al., 2019), and in experimental autoimmune encephalomyelitis (EAE) which is the most commonly used experimental model for MS (Nikić et al., 2011). Superb *in vivo* imaging studies demonstrated that abnormal mitochondria and transport deficits were already present in myelinated axons in an acute EAE model (Nikić et al., 2011; Sorbara et al., 2014). They also demonstrated that the initial swellings of the axon often occur at putative nodes of Ranvier (Nikić et al., 2011).

Similar impairment of axonal transport has been reported in knockout (KO) mice ablating PNJ proteins Caspr (Einheber et al., 1997; Bhat et al., 2001; Einheber et al., 2006) and glia-specific Neurofascin-155 (Pillai et al., 2009), in *Caspr* mutant mouse shambling (Takagishi et al., 2016), in mice ablating cytoskeletal scaffolding proteins Band 4.1B and Whirlin (Saifetiarova and Bhat, 2019), and in mice with defective synthesis of the myelin lipids ceramide galactosyltransferase (CGT)-null (Garcia-Fresco et al., 2006) and cerebroside sulfotransferase (CST)-null (Honke et al., 2002; Ishibashi et al., 2015). Furthermore, ether lipids-deficient mice (Teigler et al., 2009) and Purkinje cell-specific *Ugcg* KO mice (Watanabe et al., 2010) that displayed disrupted PNJs also showed axonal swellings. The most noticeable common finding in these PNJ-disrupted mouse models is that the focal impaired axonal transport indicated by axonal swellings has already become apparent in axons with intact compact myelin sheaths at early developmental stages (Pillai et al., 2009; Teigler et al., 2009; Ishibashi et al., 2015). Significantly, in case of CST-null mice, it has been shown that swelling occurs even if the transverse bands at the paranodes are only partially disrupted (Honke et al., 2002; Marcus et al., 2006).

Although we do not know whether the formation of PNJs is intact at early stages in acute mouse models of MS and in acute human MS lesions (Nikić et al., 2011), the observation that the initial disruption of the axon often occurs close to nodes of Ranvier seems to imply that the state of PNJs is a cause of focal axonal damage. At the paranode, the cytoplasmic portion of Caspr is associated with axonal actin cytoskeleton via scaffolding proteins Band 4.1B, Whirlin, αII and βII spectrin, and ankyrin B (Garcia-Fresco et al., 2006; Ogawa et al., 2006; Saifetiarova and Bhat, 2019). Therefore, even partial alteration of PNJs may disturb axonal cytoskeletal functions, resulting in impaired axonal transport. Taken together, the evidence suggests that the structure of PNJs is one of the most important influences on axonal transport and plays a key role as a facilitator for the transport.

## The State of PNJs and Purkinje Axonal Homeostasis

Focal axonal swellings arise in several regions of the central nervous system (CNS) in a number of neurodegenerative disorders and in traumatic brain injury (Trapp et al., 1998; Nikić et al., 2011; Sorbara et al., 2014; Jin et al., 2015; Maia et al., 2015). In these cases, the axonal swellings on cerebellar Purkinje cells—known as torpedoes—are associated with Purkinje cell loss (Louis et al., 2009, 2014; Maia et al., 2015; Ljungberg., 2016; Gionco et al., 2021). Although there are many causes of Purkinje axonal swellings, there are common morphological features in Purkinje axons in the mouse models with perturbations of PNJs, described in the following.

The Purkinje axonal swellings are usually covered with PLP-positive compact myelin in CST-null mice (Ishibashi et al., 2015). Similar observations have been made with electron microscopy (EM) analysis in other mouse models, including deletion of CGT (Garcia-Fresco et al., 2006), ether lipids (Teigler et al., 2009), glia-specific Neurofascin-155 (Pillai et al., 2007; Pillai et al., 2009), cytoskeletal scaffolding proteins Band 4.1B and Whirlin (Saifetiarova and Bhat, 2019), and *Caspr* mutant mouse shambling (Takagishi et al., 2016), suggesting that these swellings are formed in the internodal axon. In CST-null mice, the swellings along Purkinje axons became prominent only after myelin formation, and their number and size progressively increased with age (Ishibashi et al., 2015). Initially, the swellings were small and characterized by Calbindin-positive axoplasm, but gradually, axonal cytoskeletal neurofilament accumulations were observed within the swellings (Ishibashi et al., 2015). These axonal swelling features are also reported in mice ablating cytoskeletal scaffolding proteins Band 4.1B and Whirlin (Saifetiarova and Bhat, 2019). In CST-null, with the increasing size of the swellings, the accumulation of mitochondrial COXIV became prominent, followed by the detection of neurofilaments and amyloid precursor protein (APP), which is a marker for axonal damage (Ishibashi et al., 2015). The mitochondrial accumulation revealed by cytochrome c staining, as well as neurofilaments in the axonal swellings, are also reported in Caspr-null and CGT-null mice (Garcia-Fresco et al., 2006) and in Purkinje cell-specific *Ugcg* KO mice (Watanabe et al., 2010). These findings seem to indicate a process of deterioration of focal axonal transport and suggest that the focal axoplasm alteration occurs before destabilization of the axonal cytoskeleton in disrupted PNJs Purkinje axons.

Then, what could be the cause of Purkinje axonal swellings in disrupted PNJs? The most intriguing common features in Purkinje axonal swellings in mice with disrupted PNJs are accumulations of smooth endoplasmic reticulum (sER) and focal accumulations of type 1 inositol 1,4,5-trisphosphate receptor (IP_3_R1). In CST-null, EM analysis showed numerous membranous organelles, dense bodies, and tubular structures closely resembling sER in the Purkinje axonal swellings (Ishibashi et al., 2015). Similar accumulations of sER were reported in the Purkinje axonal swellings in other mutant mice with disrupted PNJs (Garcia-Fresco et al., 2006; Pillai et al., 2007; Teigler et al., 2009; Takagishi et al., 2016; Saifetiarova and Bhat, 2019), and an *in vitro* overexpression study of IP_3_R1 reported similar stacks of membranous sER (Takei et al., 1994). Furthermore, IP_3_R1 is especially abundant in Purkinje neurons and localizes to the sER (Satoh et al., 1990; Mikoshiba, 2007). All of these findings suggest that IP_3_R1 overexpression causes these sER accumulations.

Further supporting this suggestion, in CST-null mice, IP_3_R1-positive focal accumulations are the earliest finding in the Purkinje axonal swellings (Ishibashi et al., 2015), and IP_3_R1 had accumulated even in small-sized swellings that still lacked (observed) neurofilament accumulation. In the developmental stage, IP_3_R1-positive axonal swellings start appearing by 12 days of age, just after myelination in CST-null mice, and these IP_3_R1-positive axonal swellings always occur at the myelinated internode and not at the disrupted paranodal regions (Ishibashi et al., 2015). The accumulation of IP_3_R1 in the Purkinje axonal swellings was also reported in ether lipid-deficient mice (Teigler et al., 2009) and in *Caspr* mutant mouse shambling (Takagishi et al., 2016), suggesting that this is a common feature in myelinated axons with PNJs disruption. Although the molecular mechanism responsible for the focal IP_3_R1 accumulation and sER accumulation in the PNJ-disrupted Purkinje cells still need to be established, the formation of axonal swellings in disrupted PNJ Purkinje cells by accumulating IP_3_R1-rich sER may be the result of paranodal disorganization causing impaired axonal transport and disturbance of Ca^2+^ homeostasis. Therefore, the formation of a robust fence at the paranode appears to be critical for an appropriate distribution of IP_3_R1 in Ca^2+^ homeostasis in myelinated nerves.

## Discussion

It is well known that an increase in neuronal Ca^2+^ is involved in the triggering of neuronal death. Ca^2+^ release by IP_3_Rs on internal stores, such as ER and mitochondria, plays an essential role in several neurological disorders (Chiu, 2011; Barsukova et al., 2012; Takada et al., 2017), but details of these processes remain elusive. Accumulation not just of sER has been repeatedly observed in the Purkinje axonal swellings of mice with disrupted PNJs, but also of mitochondria. Because an increase in the concentration of cytosolic Ca^2+^ has been shown to lead to arrested movement of ER and mitochondria (Wang et al., 2000; Brough et al., 2005), the focal activation of IP_3_ signaling may induce alteration of motility of mitochondria as well as ER in the PNJ-disrupted Purkinje axons. Structural interactions between ER and mitochondria in mitochondria-associated ER membranes (MAMs) are crucial for Ca^2+^ transfer between ER and mitochondria (Hayashi et al., 2009; Paillusson et al., 2016). In MAMs, IP_3_R and voltage-dependent anion channel (VDAC) interact via glucose-regulated protein 75-kDa (GRP75), allowing a direct transfer of Ca^2+^ into the mitochondria (Rapizzi et al., 2002; Szabadkai et al., 2006; Paillusson et al., 2016). This suggests the involvement of a local calcium dysregulation through IP_3_R1 in the occurrence of Purkinje axonal swellings in the disrupted PNJ Purkinje cells.

A study by Yin and others revealed the importance of mitochondria-ER associations in Ca^2+^ homeostasis in myelinated axons (Yin et al., 2016), and another study by Thai and others suggests that the interactions between mitochondria and ER are enhanced in chronically demyelinated axons and maintained during axonal degeneration in hereditary myelin disease (Thai et al., 2018). To improve understanding of the relation between myelination and MAM formation and/or ER distribution in axonal Ca^2+^ homeostasis, it needs to be established whether elevated Ca^2+^ concentration occurs in the Purkinje axonal swellings of PNJ-disrupted mice. Although further experiments are needed to reveal the mechanism of accumulation of IP_3_R1, the findings from the mice with disrupted PNJs suggest that the formation of a robust fence at the paranode is critical for an appropriate distribution of IP_3_R1 in Ca^2+^ homeostasis in myelinated nerves.

The focal axonal swellings in Purkinje cells are found in various pathological conditions as well as during development. A study by Lang-Ouellette and others recently reported that Purkinje axonal swellings in healthy young mice enhance action potential fidelity and cerebellar function (Ljungberg et al., 2016; Lans-Quellette et al., 2021). However, the density of intracellular organelles in the axonal swellings did not change compared to control axons (Lans-Quellette et al., 2021), suggesting that the Purkinje axonal swellings in healthy development and disease models differ in their subcellular composition.

There are many kinds of KO and mutant mice that show disrupted PNJs, and the severity of the junctional disruption in paranodes is diverse. Therefore, such mice will be a useful tool to identify the molecular mechanisms through which myelin interacts with the axon to maintain Ca^2+^ homeostasis and to protect it from degeneration.
